# High human exposure to pyrene (polycyclic aromatic hydrocarbon) in Kinshasa, a capital of the Democratic Republic of Congo

**DOI:** 10.1186/0778-7367-71-14

**Published:** 2013-06-19

**Authors:** Joel Tuakuila, Martin Kabamba, Honoré Mata

**Affiliations:** 1 Medical and Environmental Chemistry, Faculty of Sciences, University of Kinshasa, Kinshasa, Democratic Republic of Congo; 2 Louvain Center for Toxicology and Applied Pharmacology (LTAP), Université catholique de Louvain, Avenue E. Mounier 53, box 52.02.12, 1200 Brussels, Belgium

**Keywords:** Biomonitoring, Environmental pollution, Organic compounds, Public health, Polycyclic aromatic hydrocarbons

## Abstract

**Background:**

Data on human exposure to chemicals in Africa are scarce. A biomonitoring study was conducted in a representative sample of the population in Kinshasa (Democratic Republic of Congo) to document exposure to polycyclic aromatics hydrocarbons.

**Methods:**

1-hydroxypyrene (1-OHP) was measured by HPLC fluorescence in spot urine samples from 220 individuals (50.5% women), aged 6–70 years living in the urban area and from 50 additional subjects from the sub-rural area of Kinshasa. Data were compiled as geometric means and selected percentiles, expressed without (μg/L) or with creatinine adjustment (μg/g cr). Multiple regression analyses were applied to factors (creatinine, grilled meat habits and smoking habits) influencing 1-OHP (stepwise procedure, criteria: probability F to enter ≤ 0.05 and probability F to remove ≥ 0.10).

**Results:**

According to the regression models, creatinine, grilled meat habits and smoking habits contribute to explain 45% of the variation in population’s urinary 1-OHP by the environmental exposure. Overall, living in urban area of Kinshasa was associated with increased levels of 1-OHP in urine as compared to a population living in the sub-rural area [GM: 1.8 μg/L (n = 220) versus 1.4 μg/L (n = 50), p < 0.01] as well as compared to the reference values from databases involving American or German populations.

**Conclusion:**

This study reveals the high pyrene (PAH) exposure of the Kinshasa population. However, more work, with a rigorous design in the exposed population (monitoring of air concentrations and identifying other sources of pyrene –PAH exposure), is needed to establish further documentation.

## Background

Polycyclic aromatic hydrocarbons (PAH) are produced when organic materials undergo incomplete combustion. They are composed of two or more benzene rings and occur, depending on the type of pyrolytic process and of source material, in various compounds, but always in the form of a mixture. Because so many incomplete combustion processes occur, PAH are ubiquitous environmental contaminants. Exposure to PAH is associated with lung, esophageal, gastric, colorectal, bladder, skin, prostate, and cervical cancers in human and animal models [1].

Pyrene is present in almost all PAH mixture in relatively high concentrations and there is a good correlation between pyrene and other components in PAH mixture [2]. 1-OHP (1-hydroxypyrene), a major metabolite of pyrene, has been widely used as an indicator of internal exposure to PAH [3,4].

The main source of PAH intake is food, on the one hand as a result of airborne PAH precipitating onto cereals, fruit and vegetables, and on the other hand as a result of PAH generated during the preparation of food. For example, smoked food and food grilled on open flames display substantial levels of PAH content [5].

A very important source of PAH exposure among the general population is tobacco smoke [6]. Smokers’ intake of pyrene in cigarette smoke is of the same order of magnitude as intake from average food consumption [7].

It has been shown that domestic wood burning, residential charcoal burning stoves and barbecue charcoal combustion turn out to be important sources of pollutant exposure to humans [8,9].

In DRC (Democratic Republic of Congo), only 5% of the population has access to electricity. As a result, wood energy production accounts for 85% of total energy consumption and fuel wood and charcoal are by far the most heavily consumed energy sources in DRC [10,11], used primarily for household heating and cooking.

In this study, we provide the first data for biomonitoring PAH in a representative sample of the Kinshasa population. The values were compared to those reported by the reference values from American [12] or German databases [13].

## Methods

### Study design

In the absence of reliable population registers and in view of the practical difficulties of conducting a truly random sampling in the population of Kinshasa, we applied a two-stage systematic sampling approach [14]. In the first stage, the 22 administrative entities of Kinshasa were listed in alphabetical order and 11 out of them were selected as follows: a first entity was drawn randomly from the list and every other subsequent entity was then included, thus ensuring a comprehensive coverage of the entire urban area of Kinshasa. In the second stage, we aimed to recruit about 25 healthy male and female subjects between 6 and 70 years from each of the 11 entities. In a mobilization campaign (mainly by word of mouth), healthy subjects were invited to come to the local health center to provide a urine sample. After exclusion of 13 individuals because of possible direct occupational exposure to PAH (asphalt application, waste incineration, aluminum smelting), 220 individuals provided a urine sample and were included in the present study (80% of the target number was reached). Informed consent was obtained from each subject and information on age, gender, place of residence and smoking habits were recorded. With the same methods of mobilization campaign, fifty additional subjects living in the sub-rural area of Kinshasa were also included. The characteristics of two areas (urban/sub-rural) selected: urban area had high percentage of population density; motorization, old second hand vehicles and car traffic whereas sub-rural had high percentage of green area [15]. This study was approved by the Congolese committee of medical ethics and the study results will be informed back to individuals sample donors with proper explanations.

### Laboratory methods

Great care was taken to avoid contamination during all the steps of collection, transport and analysis. Spot urine specimens were collected in metal-free polystyrene containers and stored at −20°C. The samples were then kept frozen and transported in a cool box to be analyzed by the Louvain centre for Toxicology and Applied Pharmacology (Brussels, Belgium). We determined urinary 1-OHP by HPLC (High Performance Liquid Chromatography) as described previously [16,17] with some modifications [18]. Briefly, 2.0 mL urine was used for each sample, and the identification and quantification of 1-OHP were based on retention time and peak area measured using a linear regression curve obtained from internal standard solutions. The limit of quantification (LOQ) was 0.20 μg/L. The valid urine 1-OHP concentrations were expressed as μg/L or μg/g of creatinine. The determinations of urinary cotinine (LOQ = 50 μg/L) were done by HPLC according to the methods previously described [19]. Creatinine was determined (LOQ = 0.1 g/L) on a Beckman Synchron LX 20 analyser (Beckman Coulter GmbH, Krefeld, Germany) by the Jaffe method [20]. For quality control, internal controls and reference materials were run together with the samples on a daily basis.

### Statistical analyses

Concentrations were log transformed for data analysis. Geometric means (GM), ninety-five percent confidence intervals (CI) and percentiles were calculated using NCSS version 2004 (NCSS Institute Inc. 2004). The limit of quantification (LOQ) divided by 2 was used for imputation of values lower than the LOQ [21]. Differences between samples with normal distribution were examined by the *T*-test and Chi-square test. Stepwise multiple linear regression analyses of log-transformed data were used to estimate the influence independent variables (creatinine, grilled meat habits and smoking habits) on the 1-OHP (stepwise procedure, criteria: probability F to enter ≤ 0.05 and probability F to remove ≥ 0.10). A p-value lower than 0.05 was considered as statistically significant for all tests.

## Results

Age of these 220 urban subjects was between 6 and 70 years and 31 years on average (standard deviation: 18). Most participants were adults (74.5%) and nearly half (50.5%) were female. Among adults, thirty-six percent were current smokers. The characteristics of sub-rural subjects are also presented in Table 1.

**Table 1:** Demographic characteristics of the participants

	**Urban**	**Sub-rural**	**P**
**Number of subjects**	220	50	
**Age, years**^**a**^	31 ± 18 [6–70]	36 ± 15 [6–60]	0.55
**6-14, n (%)**	56 (25.4%)	12 (24.0%)	0.83
**>14, n (%)**	164 (74.5%)	38 (76.0%)	
**Sex:**			
**Male, n (%)**	109 (49.5%)	21 (42.0%)	0.93
**Female, n (%)**	111 (50.5%)	29 (58.0%)	
**Current Smokers, n (%)**	79 (35.9%)	6 (12.0%)	**<0.01**

^a^Arithmetic mean ± SD [Range].

Geometric mean (GM) urinary 1-OHP was 1.8 μg/L (95% CI: 1.6, 2.0) (Table 2). Three (1.4%) 1-OHP measurements were less than the LOQ. As expected, smokers had higher cotinine urinary levels (Cot-U) than non-smokers [GM (95% CI): 137.3 μg/L (115.5, 163.2) versus GM (95% CI): 87.7 μg/L (70.4, 104.3)].

**Table 2:** Urinary concentrations of 1-OHP in the Kinshasa population (n = 220; 6–70 years)

			**N**	**Urinary concentrations of 1-OHP**					P*
				**Min**	**P50**	**P95**	**Max**	**GM (CI 95%)**	
Total		μg/L	220	0.1	1.6	6.6	26.8	1.8 (1.6 – 2.0)	
		μg/g cr		0.1	1.2	5.8	14.8	1.3 (1.1 – 1.4)	
Sex	Men	μg/L	109	0.2	1.6	6.5	26.8	1.6 (1.4 – 1.9)	0.19
		μg/g cr		0.2	0.9	4.7	14.8	1.0 (0.9 – 1.2)	
	Women	μg/L	111	0.1	1.8	7.6	16.4	1.9 (1.6 – 2.2)	
		μg/g cr		0.1	1.3	6.5	12.1	1.5 (1.2 – 1.7)	
Age	6 – 14 years	μg/L	56	0.4	2.1	7.6	14.7	2.1 (1.6 – 2.6)	0.10
		μg/g cr		0.3	1.7	8.9	14.8	1.9 (1.4 – 2.5)	
	> 14 years	μg/L	164	0.1	1.5	5.9	26.8	1.7 (1.5 – 1.9)	
		μg/g cr		0.1	1.0	4.7	12.9	1.1 (0.9 – 1.2)	
Smoking habits	Current smokers	μg/L	79	0.1	2.0	8.2	26.8	2.3 (1.9 – 2.7)	**<0.01**
		μg/g cr		0.1	1.2	6.3	12.9	1.3 (1.0 – 1.5)	
	Non-smokers	μg/L	141	0.2	1.6	5.2	16.4	1.5 (1.3 – 1.7)	
		μg/g cr		0.2	1.1	5.8	14.8	1.2 (1.0 – 1.4)	
Grilled meat habits	Consumers	μg/L	65	0.5	4.1	14.1	26.8	4.0 (3.4 – 4.7)	**<0.01**
		μg/g cr		0.5	2.3	12.3	14.8	2.5 (2.1 – 3.1)	
	Non-consumers	μg/L	155	0.1	1.3	2.8	6.5	1.2 (1.1 – 1.4)	
		μg/g cr		0.1	0.8	3.2	5.5	0.9 (0.8 – 1.0)	

N sample size; P50, P95 = percentiles; *Min* minimum value, *Max* maximum value;

*GM* geometric mean (CI = 95% confidence interval); *p-value (*t*-test on log-transformed values).

N < Limit of quantification (0.20 μg/L) = 3 (1.4%).

There was a statistically significant difference (p-value < 0.01) with smoking habits (0 for no/1 for yes) for 1-OHP. Age (0 for 6 – 14 years/1 for > 14 years) and sex (0 for female/1 for male) were not shown a significant difference (Table 2).

In multivariable analyses, creatinine (continuous log-variable), grilled meat habits (0 for non-consumers/1 for consumers) and smoking habits (cotinine as continuous log-variable) were the parameters significantly associated with urinary excretion of 1-OHP with 0.449 as a value of R^2^ (Table 3).

**Table 3:** Multiple regression analysis models of 1-OHP levels

**Independent variable**	**Coefficient β (95% CI)**	**Adjusted R**^**2**^
Intercept	0.415 (0.253 to 0.579)	0.449
Creatinine^a^	0.193 (0.035 to 0.349)	
Grilled meat habits^b^	0.505 (0.426 to 0.584)	
Smoking habits ^c^	0.081 (0.003 to 0.157)	

^a^Creatinine represented as continuous log-variable, ^b^Grilled meat represented as 0 for Non-consumers and 1 for consumers; ^c^ Smoking habits: cotinine represented as continuous log- variable, R^2^: explained variance (i.e. the square of the correlation coefficient). Results are given for those variables that correlated, and only when the regression was significant (p < 0.05).

## Discussion

None of the measured values of Urinary 1-OHP was significantly different among the 11 urban entities investigated; indicating that our sampling strategy “unweigted clusters” did probably not introduce a strong bias in the representativeness of our population sample.

Urinary 1-OHP, a metabolite of PAH, has been shown to be an indicator of both uptake of pyrene from foods and exposure to exogenous PAH [22]. However, an important limitation of this biomarker is that it only reflects recent exposure and tends to vary widely within individuals [23-26].

The distribution of urinary 1-OHP levels of the reference population are not Gaussian. Normalization can be obtained when expressing experimental data as a base 10 logarithm. Our results (Table 2) showed a significant difference in 1-OHP levels between current smokers and non-smokers (GM: 2.3 μg/L versus 1.3 μg/L, p < 0.01), which may be due to the fact that tobacco smoking may influence levels of urinary of 1-OHP [7,18,27-29].

**Figure 1:**
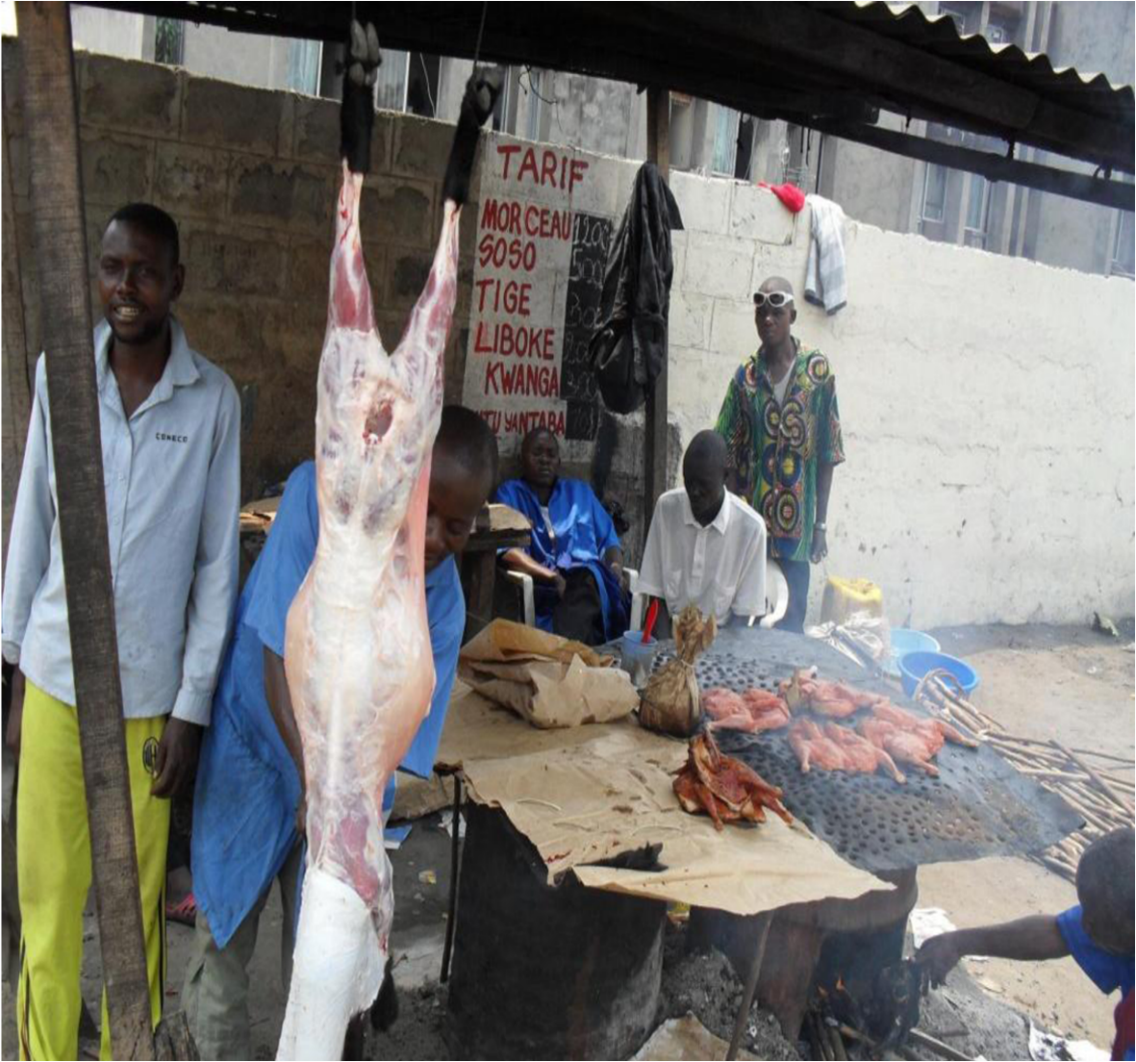
**Grilled meat habits in Kinshasa.** Source: this study: Photograph taken in September 2009.

In agreement with other studies, we found higher 1-OHP levels in consumers of grilled meat than in non-consumers (0 for non-consumers GM: 1.2 μg/L versus 1 for consumers GM: 4.0 μg/L, p < 0.01) (Figure 1; Table 2), which is not surprising since grilled meat represents an important source of PAH exposure [7,30-32].

As reported in the literature [12,33], investigations have not shown significant differences neither for sex groups nor for age groups (Table 2).

In Stepwise multivariable analyses, creatinine (continuous log-variable), grilled meat habits (yes/no) and smoking habits (continuous log-variable) were the independent parameters significantly associated with urinary unadjusted values of 1-OHP (depend parameter) with 0.45 as a value of R^2^ (Table 3).

As in other surveys, increased 1-OHP levels were measured in residents of urban areas compared to sub-rural settings [GM: 1.8 μg/L (n = 220) versus 1.4 μg/L (n = 50), p < 0.01]. The high percentage of smokers (Table 1) in the urban population could, at least partly, explain this difference.

The mean 1- OHP level in the sub-rural and the urban populations (smokers and non-smokers combined) exceeded the American and German levels (smokers and non-smokers combined) [12,13]. Children from Kinshasa were found to have much higher levels (GM [95%CI]: 2.1 μg/L [1.6-2.6] for children 6–14 years) than American children (GM [95%CI]: 0.09 μg/L [0.07-0.11] for children 6–11 years; GM [95%CI]: 0.10 μg/L [0.085-0.129] for children 12–19 years) [34]. Inhalation of emissions from charcoal burning or from cooking on open fires or traditional stoves fueled with biomass (wood, charcoal, crop and waste residues, …) either outdoor or in poorly ventilated spaces, and consumption of this broiled, smoked, fried or grilled food (Figure 1) are likely to contribute to the high levels of urinary 1-OHP in Kinshasa subjects.

The present study has several limitations. First, with regard to sample collection, selection of urinary sample donors did not follow rigid sampling strategy (such as random sampling) but by chance, which was practically inevitable under present survey conditions. Second, low number of subjects and characteristics selected. Third, passive smoking exposure is a factor affecting PAH exposure; this factor did not evaluate.

Despite such limitations, however, it is prudent to conclude that data from the present study constitutes levels generally exceeded in the Kinshasa population. Living in urban area of Kinshasa is associated with increased levels of 1-OHP in urine as compared to a reference population living in a sub-rural area of the same region. Increased levels were also found by comparison with the reference values from databases involving American or German populations.

## Conclusion

This study reveals the high pyrene (PAH) exposure of the Kinshasa population requiring the determination of PAH concentrations in ambient air of Kinshasa and limits values for the protection of human health.

## Competing interests

The authors declare that they have no competing interests.

## Authors’ contributions

JT drafted the manuscript. All authors commented the draft versions. All authors read and approved the final manuscript.
